# Development of Integrated Supportive Care Nursing Competence Scale for Cancer Survivors

**DOI:** 10.3390/healthcare12070755

**Published:** 2024-03-30

**Authors:** Eun-Jung Bae, Yun-Hee Kim

**Affiliations:** 1 College of Nursing, Catholic University of Pusan, Busan 46252, Republic of Korea; ejbae@cup.ac.kr; 2 Department of Nursing, Pukyong National University, Busan 48513, Republic of Korea

**Keywords:** competence, cancer survivor, integrated supportive care, nurse, validity and reliability

## Abstract

Nurses play a key role in providing integrated supportive care to cancer patients for their various needs. Efforts should be made to identify the competencies required for nurses providing integrated supportive care to cancer survivors, evaluate the competence level with reliable and reasonable tools, and continuously improve them. In Phase 1, the items of the scale were developed through a literature review and by conducting a focus group interview. In Phase 2, the validity and reliability of the scale were analyzed. A total of 504 nurses participated. Data were analyzed using item analysis, exploratory factor analysis, confirmatory factor analysis, Pearson’s correlation with other scales, internal consistency, and split-half reliability. The developed scale consisted of 22 items. These items were grouped into five subscales and labeled as professionalism enhancement, care coordination, comprehensive nursing needs assessment, providing tailored information and education, and recurrence surveillance and secondary cancer prevention. Confirmatory factor analysis supported good convergent and discriminant validities. The criterion validity was verified. The internal consistency of the scale measured by Cronbach’s α was 0.91. The developed scale is expected to be used as an instrument to identify cancer survivor integrated supportive care competencies of nurses in practice.

## 1. Introduction

The 5-year relative survival rate of cancer patients diagnosed in the last 5 years in Korea has increased by about 17.4% [1]. As a result, cancer survivors’ health care and quality of life after cancer treatment are attracting attention beyond the perspective of diagnosis and treatment. The definition of cancer survivor has expanded to include patients diagnosed with cancer and their families [2,3], and various physical, psychological, and social demands experienced with cancer are being recognized as social problems [4].

Cancer survivors experience side effects related to treatment, such as anorexia, nausea, vomiting, hair loss, and peripheral neuropathy, as well as late complications such as lymphedema and cognitive dysfunction [5,6,7]. After a cancer diagnosis, they complain of psychological problems such as embarrassment, sadness, fear, depression, and anxiety about metastasis and cancer recurrence [3]. Cancer survivors face difficulties in returning to work and social life due to prejudice or social stigma against cancer and the resulting economic burden [8], and they need a work and social return program that can help them overcome these difficulties [9]. They require comprehensive cancer survivor management to maintain a balance in life throughout the process, from cancer diagnosis to recovery after treatment [10]. 

In the United Kingdom, the United States, and Australia, various support programs are operated to improve the systematic management and quality of life of cancer survivors [11,12,13]. In South Korea, to improve the quality of life for cancer patients and their families, a standard management and support system has been established through the integrated support center for cancer survivors. It provides education and counseling programs centered on nurses, doctors, and social workers to address survivors’ various physical symptoms and psychosocial problems [14]. 

Nurses play a pivotal role in providing integrated supportive care to meet various needs ranging from physical, psychological, and social support needs to self-care performance, information acquisition, and lifestyle management of cancer survivors [10,15,16]. However, they showed poor performance in care for cancer survivors because of insufficient knowledge and information, unfamiliarity with care practices, low self-confidence, and a lack of competence [17,18]. Therefore, it is necessary to investigate the competence required for nurses who perform integrated supportive care and evaluate the competence level with a reliable and valid tool and continuously improve it. 

Preventing cancer recurrence and secondary cancer, providing information and education, providing emotional support, coordinating and cooperating with other fields, and demonstrating leadership are suggested as necessary competencies for nurses in providing integrated supportive care for cancer survivors [19,20,21]. Previous studies used cancer survivorship care performance scales and cancer survivor integrated supportive care competence scales to measure integrated supportive care competence for cancer survivors [22,23,24]. These scales commonly included the contents of care practice for physical and psychosocial problems following cancer treatment, monitoring for cancer recurrence, management to prevent recurrence and secondary cancer, and care coordination for cancer survivorship care practices. Additionally, while awareness, role attitude, self-confidence, and level of performance frequency have been measured for the responsibility of cancer survivorship care [22,24], the definition of attributes of the competencies required to provide care for cancer survivors is insufficient. Furthermore, the attributes of the cancer survivor supportive care competence were based on the previous literature and did not include any on-site confirmation process through an interview. Items that were not appropriate to be regarded as the integrated supportive care competence of cancer survivors by domestic nurses were included [24]. In some cases, the tool used to measure competence was not tested for reliability and validity [23,24]. 

Studies related to cancer survivor care targeting domestic nurses are scarce, except for those that investigated the practice of cancer survivor care by oncology nurses [17]. Finding a study that developed a scale to measure the cancer survivor supportive care competence of domestic nurses was difficult, and the existing scale developed to measure the care competence for cancer patients undergoing chemotherapy has limitations in evaluating the cancer survivor supportive care competence because it does not consider the aspect of care after treatment [25]. Therefore, this study aims to investigate the attributes of cancer survivor supportive care competence of nurses, develop a measurement scale, and test the validity and reliability of the developed scale.

## 2. Materials and Methods

### 2.1. Study Design 

This methodological study was conducted to develop and validate a scale to evaluate the cancer survivor supportive care nursing competence. The study was conducted according to the scale development and verification procedure presented by DeVellis [26]. 

### 2.2. Phase 1. Development of the Scale 

In the scale development stage, preliminary items were formed through a systematic literature review and focus group interview.

#### 2.2.1. Systematic Literature Review

In order to compose preliminary items, domestic and foreign literature on cancer survivor supportive care competence was reviewed. A literature search was performed with similar words using keywords such as “cancer”, “survivor”, “integrated care”, and “nurse”. The search period was set from 2000 to 2019, when discussions on cancer survivors were actively being conducted. Twenty-two studies selected through the literature search and selection/exclusion process were reviewed (Supplemental Material S1). 

#### 2.2.2. Focus Group Interview

The focus group interview participants included nurses who are currently working in hospitals and have more than 5 years of experience caring for cancer patients. Participants were recruited through the snowball sampling method and included 13 nurses. Three focus groups were formed with four–five participants per group, and each group was interviewed once for 60–120 min. The main questions were as follows: “What do you think integrated supportive nursing for cancer survivors is?” and “What knowledge, attitudes, and skills do you think nurses should have when caring for cancer survivors?” Data analysis was conducted according to the content analysis method suggested by Elo and Kyngäs [27]. 

#### 2.2.3. Preliminary Items

The commonality between the literature review and focus group interview data was identified. A comprehensive analysis was conducted focusing on whether it was an important concept corresponding to the cancer survivor integrated supportive care competence. Preliminary items were extracted by dividing the areas. The content validity of the preliminary questions was evaluated by a panel of experts comprising two nursing professors with research experience related to nurses or cancer survivors, three nurses specializing in oncology, and four nurses with over 8 years of experience in caring for cancer patients. The relevance of the developed preliminary items to cancer survivor integrated supportive care competence was evaluated on a 4-point scale (4 points for “strongly agree”, 3 points for “agree”, 2 points for “disagree”, and 1 point for “strongly disagree”). Items with an Item–Content Validity Index of 0.80 or higher were selected [28]. The contents were revised according to the opinions of experts on duplicated items, difficult-to-understand parts, and parts that needed to be modified in terms of expression. In addition, advice was taken from a Korean literature major about awkward expressions, problems with grammar, and the overall flow of sentences; accordingly, word changes, position adjustments, and spacing and postposition corrections were made. 

A preliminary survey was conducted to check the understanding of the developed scale and the time to fill out the questionnaire, and to find and correct errors in the application of the scale. A preliminary survey was conducted with 25 nurses working in cancer wards of specialized cancer hospitals and tertiary general hospitals. 

### 2.3. Phase 2. Evaluation of the Scale

The developed scale was evaluated in terms of its validity and reliability.

#### 2.3.1. Participants and Data Collection 

A survey was conducted from 2 April to 30 May 2020 to evaluate the validity and reliability. The participants were nurses in charge of caring for cancer patients for over 3 months and were recruited through the cooperation of 7 hospitals in Korea. A face-to-face survey was conducted on nurses who understood the contents of the questionnaire and agreed to participate. The absolute sample size for exploratory factor analysis was at least 100 people [29]. Furthermore, several cases ranging from approximately 200 to 400 were considered appropriate for confirmatory factor analysis in constructing a structural equation model [29]. As construct validity and model fit would be exaggerated upon performing exploratory and confirmatory factor analyses on the same subject [30], 250 people were planned for each factor analysis, and 530 copies of the questionnaire were distributed, considering a dropout rate of 10%. Data were collected from 521 people (recovery rate of 98.3%), and the data of 504 people, excluding 17 copies with insufficient contents, were allocated for exploratory (250) and confirmatory (254) factor analyses through random sampling using the SPSS/WIN 25.0 program.

#### 2.3.2. Research Scales

To evaluate the concurrent validity, one of the criterion validity of the developed scale, the cancer survivorship care confidence scale was used after obtaining consent from the author. This scale was prepared by supplementing and modifying the cancer survivorship care practice scale developed by Wallace et al. [24] and evaluating it for content validity. It consists of 29 items with attributes similar to those of the developed scale. The items are scored on a 10-point scale, with 0 points for “no confidence”, 4–6 points for “moderate confidence”, and 9–10 points for “high confidence”. In the study by Kim et al. [17], Cronbach’s α of the scale was 0.97.

#### 2.3.3. Data Analysis

The collected data were analyzed using SPSS/WIN 25.0 and AMOS 21.0. Descriptive statistics were used for the general characteristics of the participants, and construct validity was tested by item analysis, exploratory factor analysis, and confirmatory factor analysis. 

For the item analysis, items with skewness and kurtosis values less than ±2.0, floor effect and ceiling effect less than 30%, and item-total correlation coefficient greater than 0.30 were selected. Kaiser–Meyer–Olkin and Bartlett’s sphericity tests were performed to examine the suitability of the exploratory factor analysis, and factors were extracted by principal component analysis by Verimax orthogonal rotation. Appropriate factors were extracted through the criteria of a Scree plot, an eigenvalue of 1.0 or more, and a total explanatory variance of 60% or more, and factor analysis was repeated by sequentially removing items with a factor loading value of 0.40 or less for each item, items with cross factor loading or a difference in loading values between factors showing a cross factor loading value of less than 0.20 [29], and items loaded differently from the theoretical basis.

For confirmatory factor analysis, maximum likelihood estimation was used. The goodness of fit of the model was evaluated by checking the Chi-square used as an absolute fit index, standard Chi-square, standardized root-mean-square residual, root-mean-square error of approximation, the Tucker–Lewis index used as an incremental fit index, and the Comparative Fit Index [15,31]. The criteria that the standardized factor loading for each item to explain the factor should be at least 0.50 and less than 0.95 and that the number of items for each factor should be three or more, in general, were applied [32]. Furthermore, if the negative error variance of the estimation error was larger than the limit value, the corresponding error term appeared negative (-), resulting in a Heywood case in which the analysis result was unreliable [33].

For convergent validity, standardized factor loadings of 0.50–0.95, average variance extracted of 0.50 or higher, and construct reliability of 0.70 or higher were applied [32,34]. Discriminant validity was tested based on whether the was greater than the square of the correlation coefficient and whether the correlation coefficient ± (2 × standard error) value did not contain 1 [32]. The criterion validity confirmed Pearson’s correlation coefficient with the cancer survivorship care confidence scale.

The reliability of the scale was confirmed through Cronbach’s α of each factor and total score, the Spearman–Brown coefficient, and the Guttman split-half coefficient. Cronbach’s α of 0.70 or higher was considered reliable [35], and 0.80–0.90 was evaluated as the best reliability [26]. 

### 2.4. Ethical Considerations

For the ethical consideration of the participants, approval was obtained from the Institutional Review Board of Dongnam Institute of Radiological and Medical Sciences (D-1912-006-002, D-2004-014-002). After explaining the study purpose during focus group interviews and surveys, data collection was carried out only for those who understood it and voluntarily agreed to participate in writing. Furthermore, details on the researcher and research topic were provided. Participants were informed about the confidentiality of their data and the voluntary nature of participation. The anonymity of the collected data was guaranteed, and they were informed that the contents would not be used outside of research purposes. They were also informed that all data collected during the research process would be stored in a locked place, secured by setting a computer password and permanently deleted and destroyed after being stored for 3 years from the end of the study.

## 3. Results

### 3.1. Phase 1. Development of the Scale

Based on the final analysis of the literature review and the focus group interview, the cancer survivor supportive care competence was divided into individual and organizational aspects. The individual aspect included assessing cancer survivors’ nursing needs in many ways and providing the interventions that they needed; it also included effective communication, critical thinking, smooth interpersonal relationships, education, and professional development. The organizational aspect included the ability to manage the integrated supportive care system for cancer survivors and exhibit leadership. 

In total, 82 preliminary items were extracted, and 17 items with an Item–Content Validity Index of less than 0.80 were deleted after the content validity evaluation. Additionally, 68 items were prepared based on the opinion that dividing the item “Respect the rights of patients in nursing and decision-making, and if necessary, request cooperation from the Ethics Committee and the Ministry of Justice” into two would be better.

As a result of the preliminary survey, no item was found to be difficult to understand. The font size and length of each item were evaluated as appropriate, and the average duration of the survey was 14 min.

### 3.2. Phase 2. Evaluation of the Scale

#### 3.2.1. General Characteristics of Participants

A total of 504 participants were included, and 96.2% of them were females. The average age was 29.76 years, the highest level of education was a bachelor’s degree with 76.0%, and the most common position was that of a general nurse with 95.8%. The total clinical experience was 7.3 years on average, with 37.1% or more having 7 years of experience or more. The average nursing experience of cancer patients was 4.7 years, with most of them (29.6%) having 1–3 years of experience. Furthermore, 87.1% had no prior experience in nursing education related to cancer survivors (Table 1).

#### 3.2.2. Item Analysis

The mean, standard deviation, minimum and maximum values, and skewness and kurtosis of each item were checked. Items with skewness and kurtosis of ±2.0 or higher were considered modified items [36]. The skewness and kurtosis of each item did not exceed the standard values. After evaluating whether the frequency (%) of the item with the lowest score and that with the highest score through the floor effect and ceiling effect was less than 30.0% of the standard value [37], seven items were deleted. As no items had a modified item-total correlation coefficient of less than 0.30, no additional items were deleted. 

#### 3.2.3. Construct Validity Evaluation

##### Exploratory Factor Analysis

Considering scale selection and ease of use [26], as well as consequential validity to prevent unintended results, the 61 items were reviewed by one professor of nursing, one nurse specializing in oncology, and three nurses with over 7 years of cancer patient nursing experience to see if there were overlapping attributes or items requiring correction of the scale. The preliminary items were rearranged into 26 items by reflecting the opinions of experts. Exploratory factor analysis was conducted using principal component analysis and Varimax orthogonal rotation. After the first exploratory factor analysis, two items were deleted as they overlapped and belonged to two factors, and the difference in factor loadings between factors was less than 0.20. After the secondary exploratory factor analysis, two items were found to be one factor each, so these were deleted as well. The third exploratory factor analysis revealed a KMO value of 0.88, which was close to 1, indicating a common latent factor between the items. The results of Bartlett’s sphericity test (χ^2^ = 2149.18, *df* = 231, *p* < 0.001) were found to be statistically significant, confirming that the data were suitable for factor analysis. All items had a factor loading value of 0.40 or higher, and no item showed redundant factor loadings. The number of factors was set to 5 based on the case where the elbow point was 5 in the screen diagram, and the eigenvalue was greater than the reference value of 1.0. According to the criterion that the cumulative explanatory variance should be 60.0% or more, the cumulative explanatory variance of the five factors is 60.11%, which satisfied the criterion. The explanatory variance ratio of each factor is 16.13% for Factor 1, 12.79% for Factor 2, 11.04% for Factor 3, 10.77% for Factor 4, and 9.40% for Factor 5. It was evenly distributed without being biased by any particular factor.

Each factor is named as follows. Factor 1 is named “professionalism development”, Factor 2 is named “care coordination”, Factor 3 is named “comprehensive nursing needs assessment”, Factor 4 is named “providing tailored information and education”, and Factor 5 is named “recurrence surveillance and secondary cancer prevention” (Table 2).

##### Confirmatory Factor Analysis

The maximum likelihood method was used for the estimation of confirmatory factor analysis. The goodness-of-fit analysis indicated that the model was not suitable with χ^2^ of 384.46 (*df* = 199, *p* < 0.001), but other absolute fit indices indicated that χ^2^/*df* was 1.93, standardized root-mean-square residual was 0.05, and root-mean-square error of approximation was 0.06, thereby indicating that the model was suitable. As for the incremental fit indices, the Tucker–Lewis index and Comparative Fit Index are 0.89 and 0.91, respectively, which are close to the values suggested by Hair et al. [29]. Thus, the various indices indicate that the cancer survivor integrated supportive care competence scale for nurses has a good fit.

Confirmation of the standardized factor loadings for 22 items of 5 factors in the confirmatory factor analysis indicated a value of 0.45 or more and 0.85 or less, and all items except for No. 4 (0.47) and No. 6 (0.45) were above the standard value of 0.50. Items 4 and 6 were important for assessing the care needs of cancer survivors, with Item–Content Validity Index scores of 1.00 and 0.89, respectively, which were evaluated as appropriate by clinical experts. Hence, they were not deleted. There was no Heywood case in which the number of observed variables for latent variables was three or more, and the error term was negative [32]. 

#### 3.2.4. Convergent Validity and Discriminant Validity Evaluation

The average variance extracted value for each factor of the developed tool is 0.53–0.69, meeting the standard value of 0.50 or higher, and the concept reliability is 0.84–0.91, meeting the standard value of 0.70 or higher, indicating convergent validity (Table 3). Discriminant validity was tested based on whether the average variance extracted was greater than the square of the correlation coefficient and whether the correlation coefficient ± (2 × standard error) value did not contain 1 [32]. In this study, the average variance extracted value was 0.53–0.69, the square value of the correlation coefficient between the sub-factors was 0.27–0.69, and the average variance extracted value, except for the relationship between Factors 2 and 4, was greater than the squared value of the correlation coefficient between sub-factors. Furthermore, the discriminant validity, which was verified as the correlation coefficient ± (2 × standard error) value, does not include 1 (Table 4). 

#### 3.2.5. Criterion Validity Evaluation

In this study, to test criterion validity, the correlation between the developed scale and the cancer survivorship care confidence scale was confirmed. Consequently, the value obtained is 0.50, and all five factors have a statistically significant positive correlation (*p* < 0.001) (Table 5).

#### 3.2.6. Reliability

Internal consistency reliability indicated by Cronbach’s α is 0.91, and by sub-factor, it is 0.82 for professionalism development, 0.80 for partnership, 0.73 for comprehensive nursing needs assessment, 0.77 for education, and 0.76 for providing tailored information. Because Cronbach’s α for each of the final items and factors meets the standard value of 0.70 or higher, reliability is secured. As for the split-half reliability, Cronbach’s α is 0.80 for odd numbers and 0.82 for even numbers. The correlation between items is 0.88, the Spearman–Brown coefficient is 0.94, and the Guttman split-half coefficient is 0.94, showing a high correlation. Therefore, as each item represents the same concept, the homogeneity of the scale is secured.

## 4. Discussion

The study was conducted based on the scale development procedure suggested by Devellis [26]. In previous studies, only the relevant literature was considered in developing scale items [23,24]. However, this study constructed preliminary items, including the attributes of integrated supportive care competence recognized by nurses associated with the care of cancer survivors, through focus group interviews and a literature review. When developing a scale to measure nursing competency, securing its validity and reliability and considering whether it can be used in the actual field is necessary [38]. In this regard, the scale developed in this study is significant because it was developed by exploring the attributes recognized by domestic nurses, and its usability in the domestic environment was verified. The number of items was 22, similar to that in the scales presented in previous studies [22,23,24]. However, as each item seemed somewhat lengthy compared with other scales, further research may be necessary to revise or supplement the item contents and re-examine them.

Furthermore, the scale’s first factor, “professional development”, showed the highest explanatory power in factor analysis. Nurses voluntarily participate in educational and research activities and develop their expertise to provide quality, integrated, supportive care to cancer survivors. These competencies have also been suggested in previous studies [20,25]. Nurses are required to acquire the professional knowledge and skills necessary for cancer survivor integrated supportive care and provide the latest nursing intervention by integrating valid and grounded information.

The second factor, “care coordination”, can be seen as two or more people working together to share professional skills and improve the care quality. Forming a cooperative relationship with other healthcare providers is an important factor in effectively addressing the unmet nursing needs of cancer survivors. Additionally, supporting the autonomy of cancer survivors and their families and encouraging them to participate actively in healthcare leads to cooperation in cancer survivor integrated supportive care and positively affects self-management [39]. In the long term, it can sustain behavior change [40]. Care coordination is one of the common elements of cancer survivor integrated supportive care suggested in previous studies [20], and is a key attribute included in all scales used in previous studies [22,23,24].

The third factor was “comprehensive nursing needs assessment”. Cancer survivors have multiple and complex nursing needs. Nurses should be able to fully assess and evaluate these needs to provide integrated supportive care competence. Comprehensive nursing needs assessment competence was similar to the resource assessment competence presented by Smith and Lichtveld [21] and the high-risk group assessment competency presented by Macmillan Cancer Support [20], but it covered a wider range of applicable subjects or areas. Furthermore, cancer survivors want healthcare workers to ask about and confirm their physical and psychological needs, which healthcare workers often fail to assess systematically or capture accurately in practice [41]. Therefore, nursing competence is required to accurately identify their nursing needs and provide integrated supportive care.

The fourth factor was “providing tailored information and education”. Nurses should be able to help cancer survivors manage their health and the symptoms and side effects associated with diagnosis and treatment to maintain the best quality of life in daily life [42]. Nurses who provide cancer survivor integrated supportive care competence should be able to effectively use communication skills and scales to evaluate survivors’ information and educational needs and provide tailored information and education considering the type of cancer, treatment method, age, etc. Additionally, family support is a major factor that helps survivors perform self-management [43], so interventions involving families are expected to be highly effective. This ability to provide tailored information and education has been suggested in several previous studies as a component of the integrated supportive nursing care competency, so it can be said to have satisfied theoretical validity [44].

The fifth factor was “recurrence surveillance and secondary cancer prevention”. Cancer survivors live with the risk of disease recurrence and are at a higher risk than the general population of developing new secondary cancer, regardless of the first diagnosis. Therefore, monitoring cancer recurrence through periodic hospital visits and check-ups and practicing health behaviors to prevent secondary cancer is necessary. Significantly, these survivors understand that regular follow-up screenings will take care of any health problems, but only approximately 40% of them are correctly screened for secondary cancer [45]. Furthermore, 33% of male survivors continue smoking, and 76% continue drinking alcohol, thus requiring management [46]. Therefore, the ability of nurses to recognize the importance of cancer survivors performing regular check-ups, practicing health behaviors, and managing them is crucial in terms of their lifelong health management. These attributes are consistent with those of the cancer survivor integrated supportive care competence scale presented in previous studies [22,23,24].

The confirmatory factor analysis for testing construct validity used data other than those used for the exploratory factor analysis and could be seen as confirming validity based on strict standards. Consequently, standardized root-mean-square residual, root-mean-square error of approximation and the Comparative Fit Index met the goodness-of-fit criteria, but the Tucker–Lewis index did not. A construct validity test that evaluates a model by relying only on a single fit index is undesirable, and because multiple fit indices must be considered together, a measurement model was adopted in this study.

In this study, convergent and discriminant validities were secured, and criterion validity was confirmed using previous scales. The “cancer survivorship care confidence” scale selected as a criterion in this study was developed to measure cancer survivors’ confidence in nursing practice, and the correlation value with the scale developed in this study was 0.50. This satisfied the criterion that a correlation of 0.40–0.80 should be shown to secure criterion validity [35]. However, the correlation value with the sub-factors of the scale was generally low at 0.30–0.48. The criterion validity scale seemed to be presented for the cancer survivor care practice of nurses, but the correlation was low as it did not sufficiently reflect the components of care competence, including attitude, value, and performance for practice. In this regard, additional research is needed to confirm the correlation with this scale by adding a scale that can check the predictive validity.

The developed scale included professional development factors that could not be measured in the scales used in previous studies [22,24], and it was different in that it considered the ability necessary for nursing practice rather than the nursing practice itself. While existing scales comprised those made for research and whose criterion validity or reliability test results have not been confirmed, this study had the advantage of testing construct and criterion validities and reliability. Therefore, unlike existing scales, this scale can comprehensively measure the cancer survivor integrated supportive care competence of nurses, and it can be presented as a scale with verified validity and reliability that can be used in practice.

As a limitation of this study, no sample was collected considering the nursing careers related to cancer patients by cancer type or the education experience related to integrated supportive nursing for cancer survivors in the scale evaluation stage. Additionally, it did not suggest a cut-off point due to the absence of an absolute standard for cancer survivor integrated supportive care competence.

Based on this study, it is necessary to re-evaluation the validity and reliability of the scale for nurses working in environments where cancer survivors with experience in treatment are likely to visit. In addition, follow-up studies are needed to determine variables that measure the results of cancer survivor integrated supportive care competence in nurses and to present cut-off points.

## 5. Conclusions

Construct validity of the cancer survivor integrated supportive care competence scale for nurses developed in this study was confirmed through exploratory and confirmatory factor analyses, and criterion validity was verified through correlation with existing scales. The evaluation of internal consistency reliability and split-half reliability indicated that the reliability coefficient was higher than the standard value; thus, the scale’s consistency was secured. Through this process, a self-report questionnaire consisting of five factors and 22 items was established, scored on a 5-point scale from 1 point for “strongly disagree” to 5 points for “strongly agree”.

The factors of cancer survivor integrated supportive care competence of nurses derived from this study can provide guidelines regarding the competencies required for nurses in nursing practice. The developed scale can be effectively used to evaluate nurses’ integrated supportive care competency for cancer survivors and to develop competency-oriented educational programs and evaluate their effectiveness. In addition, the results of this study can serve as an opportunity to inform the importance of enhancing the integrated supportive care competency of nurses for cancer survivors and are expected to contribute to the activation of integrated supportive care for cancer survivors.

## Figures and Tables

**Table 1:** Characteristics of participants. (N = 504).

Variable	Category	n (%)/Mean ± SD
Gender	Female	485 (96.2%)
	Male	19 (3.8%)
Age	(Year)	29.76 ± 6.7
Educational	Diploma	68 (13.5%)
level (graduation)	Bachelor’s	383 (76.0%)
	Above master’s	53 (10.5%)
Position	Head nurse	21 (4.2%)
	Staff nurse	483 (95.8%)
Working place	Ward	465 (92.3%)
	Outpatient	39 (7.7%)
Experience in nursing	(Year)	7.3 ± 7.0
	<1	35 (6.9%)
	1–3	125 (24.8%)
	3–5	93 (18.5%)
	5–7	64 (12.7%)
	≥7	187 (37.1%)
Experience in nursing care for	(Year)	4.7 ± 4.8
cancer patients	<1	81 (16.1%)
	1–3	149 (29.6%)
	3–5	97 (19.2%)
	5–7	57 (11.3%)
	≥7	120 (23.8%)
Education experience in nursing care for	Yes	92 (18.3%)
cancer survivors	No	412 (81.7%)

SD = standard deviation.

**Table 2:** Exploratory factor analysis. (N = 250).

Item (I Can ...)		Factor				
		1	2	3	4	5
61	Contribute to the advancement of integrated supportive nursing care for cancer survivors by participating in relevant continuing education, license acquisition, publication, conferences, and presentations	0.756	0.126	0.153	−0.111	−0.024
66	Apply nursing intervention based on newly discovered grounds by identifying the latest trends in integrated supportive nursing care for cancer survivors.	0.741	0.024	0.021	0.198	0.177
52	Identify and manage the factors hindering or promoting integrated supportive nursing care for cancer survivors.	0.713	0.054	0.207	0.272	0.077
51	Improve the quality of nursing by evaluating the integrated supportive nursing care provided to cancer survivors.	0.707	0.247	0.108	0.240	0.016
53	Define the performance goals of integrated supportive nursing care for cancer survivors.	0.663	0.203	0.189	0.221	0.024
35	Cooperate with colleagues, doctors, and other healthcare providers to continuously provide integrated, supportive nursing care for cancer survivors.	0.186	0.725	0.271	0.069	0.164
34	Provide non-discriminating nursing care by respecting social and cultural diversities such as beliefs, customs, ethnicities, and religions of cancer survivors and families.	0.239	0.720	0.155	−0.052	0.125
39	Encourage cancer survivors and their families to express their demands and actively participate in management for recovery.	0.108	0.718	0.115	0.251	0.160
37	Treat cancer survivors with a receptive attitude.	0.153	0.624	0.084	0.413	0.035
41	Support autonomy and self-determination of cancer survivors.	0.014	0.620	0.006	0.348	0.035
2	Assess the physical symptoms of cancer survivors after cancer treatments.	0.066	0.115	0.823	0.142	0.035
1	Collect information on cancer diagnosis, treatment process, and follow-up management of cancer survivors.	0.180	0.128	0.760	0.144	0.054
3	Assess the psychological state of cancer survivors after treatment.	0.107	0.194	0.631	0.239	0.178
4	Assess the socioeconomic state of cancer survivors after treatment.	0.382	0.091	0.453	−0.064	0.135
6	Assess the supportive care demands of cancer survivors using a verified evaluation tool.	0.418	0.055	0.445	−0.027	0.177
21	Apply proper verbal and non-verbal communication skills to understand the supportive care demands of cancer survivors.	−0.014	0.320	0.157	0.673	0.246
23	Select and use the most effective method for communicating with cancer survivors.	0.222	0.133	0.226	0.656	0.219
45	Verify if cancer survivors had accurately understood the education content and provide relevant feedback.	0.419	0.227	0.086	0.606	0.078
44	Provide personalized education by considering the demand for education, literacy, language, and cultural influence of cancer survivors and their families.	0.457	0.232	0.108	0.548	−0.020
15	Explain to cancer survivors the necessity of periodic visits to the hospital and examinations to inspect the recurrence or metastasis of cancer.	0.003	0.095	0.172	0.158	0.818
13	Explain to cancer survivors the necessity of regular cancer screening for the early detection of new cancers.	0.050	0.131	0.120	−0.015	0.799
16	Suggest to cancer survivors strategies and behaviors necessary to prevent diseases and improve health.	0.219	0.167	0.041	0.307	0.672
Eigenvalue		3.55	2.81	2.43	2.37	2.07
Variance (%)		16.13	12.79	11.04	10.77	9.40
Cumulative variance (%)		16.13	28.91	39.95	50.71	60.11
Kaiser–Meyer–Olkin values: 0.88. Barlett’s sphericity test: *p* < 0.001						

**Table 3:** Factor loading and convergent validity in confirmatory factor analysis. (N = 254).

Factor	Item	Estimate		SE	CR	SMC	AVE	Construct Reliability
		B	β					
Professionalism development	53	1.00	0.74			0.55	0.58	0.87
	51	0.95	0.69	0.10	9.94 ***	0.47		
	52	0.87	0.69	0.09	9.98 ***	0.48		
	66	0.87	0.64	0.09	9.32 ***	0.41		
	61	0.81	0.54	0.10	7.90 ***	0.30		
Care coordination	41	1.00	0.66			0.43	0.66	0.91
	37	0.99	0.63	0.12	8.44 ***	0.40		
	39	1.14	0.72	0.12	9.36 ***	0.52		
	34	1.10	0.64	0.13	8.53 ***	0.41		
	35	1.05	0.68	0.12	8.95 ***	0.46		
Comprehensive nursing needs assessment	6	1.00	0.45			0.20	0.53	0.84
	4	0.96	0.47	0.18	5.21 ***	0.22		
	3	1.26	0.71	0.20	6.39 ***	0.51		
	1	1.24	0.61	0.21	5.99 ***	0.37		
	2	1.41	0.74	0.22	6.48 ***	0.55		
Providing tailored information and education	44	1.00	0.60			0.36	0.62	0.87
	45	1.15	0.69	0.13	8.56 ***	0.48		
	23	1.05	0.65	0.13	8.18 ***	0.42		
	21	1.09	0.69	0.13	8.54 ***	0.47		
Recurrence surveillance and secondary cancer prevention	16	1.00	0.71			0.51	0.69	0.87
	13	1.00	0.64	0.11	9.12 ***	0.41		
	15	1.42	0.85	0.13	11.17 ***	0.72		
Model fitness: χ^2^ = 384.46 (*p* < 0.001), χ^2^/*df* = 1.93, SRMR = 0.05, RMSEA = 0.06, TLI = 0.89, CFI = 0.91								

B = regression weights; β = standardized regression weights; SE = standard error; CR = critical ratio; SMC = squared multiple correlations; AVE = average variance extracted; SRMR = standardized root-mean-square residual; RMSEA = root-mean-square error of approximation; LO = low; HI = high; TLI = Tucker–Lewis index; CFI = Comparative Fit Index; *** *p* < 0.001.

**Table 4:** Discriminant validity in confirmatory factor analysis. (N = 254).

Factor Name	Φ^2^				AVE
	Factor 1	Factor 2	Factor 3	Factor 4	
Professionalism development	1				0.58
Care coordination	0.27	1			0.66
Comprehensive nursing needs assessment	0.44	0.33	1		0.53
Providing tailored information and education	0.56	0.69	0.52	1	0.62
Recurrence surveillance and secondary cancer prevention	0.36	0.39	0.51	0.54	0.69
Criteria	AVE > Φ^2^				
**Factor A↔Factor B**		**Φ**	**SE**	**Φ − 2 × SE**	**Φ + 2 × SE**
Factor 1↔Factor 2		0.52	0.04	0.47	0.56
Factor 1↔Factor 3		0.67	0.05	0.62	0.72
Factor 1↔Factor 4		0.75	0.05	0.69	0.80
Factor 1↔Factor 5		0.60	0.05	0.55	0.65
Factor 2↔Factor 3		0.57	0.04	0.54	0.61
Factor 2↔Factor 4		0.83	0.05	0.79	0.88
Factor 2↔Factor 5		0.62	0.04	0.58	0.67
Factor 3↔Factor 4		0.72	0.04	0.68	0.77
Factor 3↔Factor 5		0.71	0.05	0.66	0.76
Factor 4↔Factor 5		0.74	0.05	0.69	0.79
Criteria	Whether [Φ ± 2 × SE] includes 1.0				

Φ^2^ = squared correlation; Φ = correlation; AVE = average variance extracted; SE = standard error.

**Table 5:** Correlation between the developed scale and the cancer survivor nursing confidence scale. (N = 254).

	Factor 1	Factor 2	Factor 3	Factor 4	Factor 5	Total
**Cancer survivor nursing confidence**	0.38 ***	0.30 ***	0.48 ***	0.42 ***	0.37 ***	0.50 ***

*** *p* < 0.001.

## Data Availability

Data are contained within the article and supplementary materials.

## Supplementary Materials

The following supporting information can be downloaded at: https://www.mdpi.com/article/10.3390/healthcare12070755/s1, S1: Literature search strategies and PRISMA flow chart for selection of included studies, S2: Final version of the scale.
