# Supplementary material for: Development of Integrated Supportive Care Nursing Competence Scale for Cancer Survivors
Source: Healthcare (Basel). 2024 Mar 30;12(7):755. doi: 10.3390/healthcare12070755 (PMC11011853; doi:10.3390/healthcare12070755)
Supplement: Supplementary file 1 [file healthcare-12-00755-s001.zip › healthcare-2884042-supplementary.pdf]

# Supplementary material S1 ; Literature search strategies and PRISMA flow chart for selection of included studies

## 1. Literature search strategies

1) Search engines : Four global search engines in English (PubMed, CINAHL, EMBASE, Web of Science) and three domestic search engines in Korean (RISS, KoreaMED, DBpia).

2) Search terms combination :

("neoplasms [MeSH/Emtree]" OR "neoplasm*" OR "cancer" OR "tumor" OR "tumour" OR "carcinoma" OR "malignan*" OR "oncology") AND ("survivors [MeSH/Emtree]" OR "survivor*" OR "survivorship") AND ("comprehensive health care [MeSH]" OR "comprehensive health care" OR "comprehensive care" OR "comprehensive service" OR "supportive care" OR "supportive service" OR "support" OR "integrated care" OR "integrated service") AND ("nurses [MeSH/Entree]" OR "nurse*" OR "nurse practitioners [MeSH/Emtree]" OR "nurse Practitioner*" OR "nurse specialists [MeSH/Emtree]" OR "nurse specialist*" OR "nurse's role [MeSH]" OR "nurse's role" OR "nurse's practice Patterns [MeSH]" OR "nurse's practice Patterns" OR "nurse-led" OR "nurse-led service" OR "nursing [MeSH/Emtree]" OR "nursing" OR "nursing intervention" OR "nursing staff [MeSH/Emtree]" OR "nursing staff" OR "oncology nursing [MeSH]" OR "oncology nursing" OR "cancer nursing")

* Filters: Publication date from 2000/01/01 to 2019/12/31; all adult

## 2. PRISMA flow chart for selection of included studies

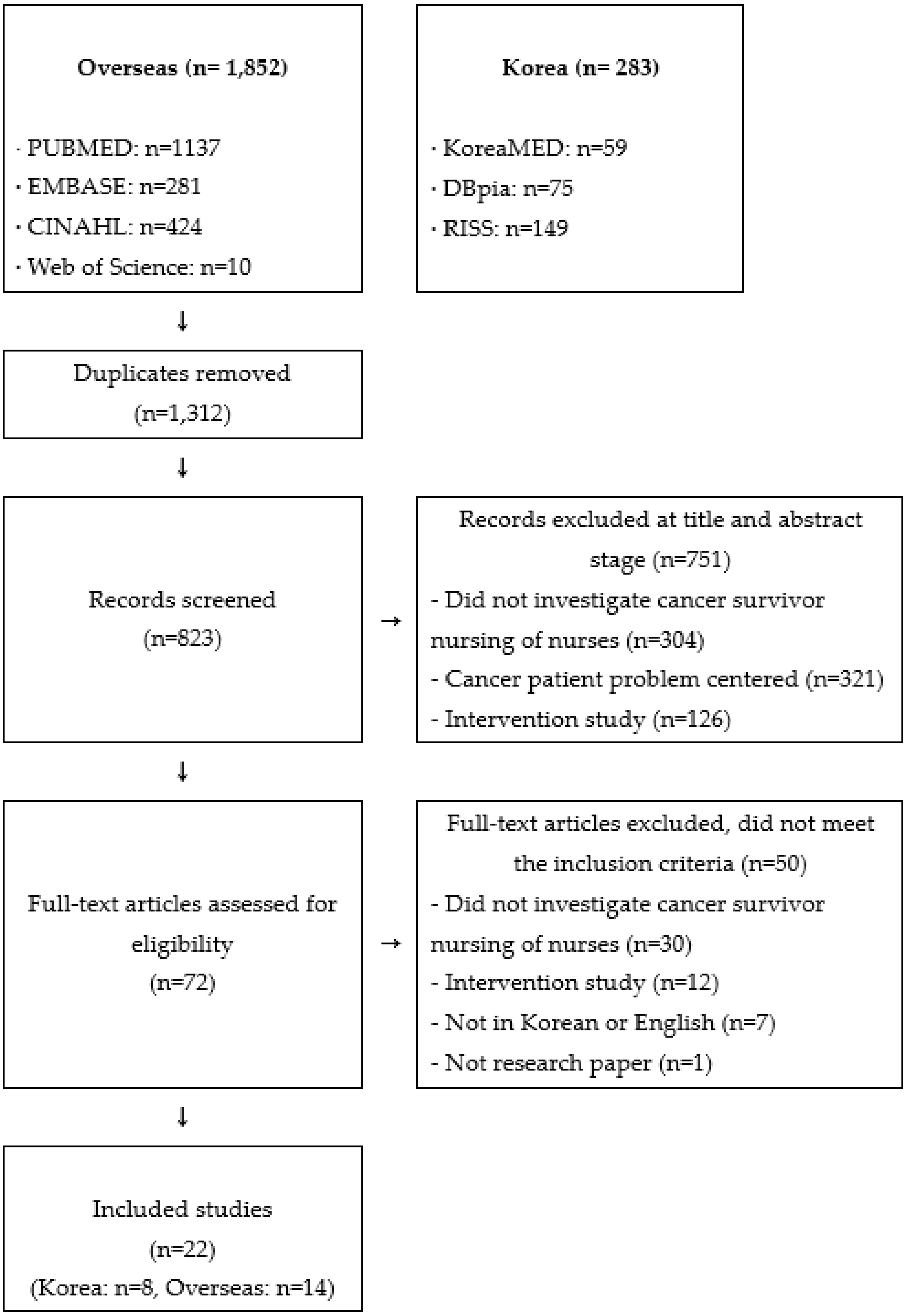

## Supplementary material S2 ; Final version of the scale

**Integrated Supportive Care Nursing Competence Scale for Cancer Survivors:**

**(English Translated)**

This scale is a self-report questionnaire consisting of five factors and 22 items, scored on a 5-point scale from 1 point for "strongly disagree" to 5 points for "strongly agree."

※ The following is about the integrated supportive care nursing competence for cancer survivors. Please mark the √ for each question closest to your actions or thoughts.

(1 = strongly disagree, 2 = agree, 3 = neutral, 4 = agree, 5 = strongly agree)

| ( I can ...) | | 1 | 2 | 3 | 4 | 5 |
|---|---|---|---|---|---|---|
| 1 | Collect information on cancer diagnosis, treatment process, and follow-up management of cancer survivors. | | | | | |
| 2 | Assess the physical symptoms of cancer survivors after cancer treatments. | | | | | |
| 3 | Assess the psychological state of cancer survivors after treatment. | | | | | |
| 4 | Assess the socioeconomic state of cancer survivors after treatment. | | | | | |
| 5 | Assess the supportive care demands of cancer survivors using a verified evaluation tool. | | | | | |
| 6 | Explain to cancer survivors the necessity of periodic visits to the hospital and examinations to inspect recurrence or metastasis of cancer. | | | | | |
| 7 | Explain to cancer survivors the necessity of regular cancer screening for the early detection of new cancers. | | | | | |
| 8 | Suggest to cancer survivor's strategies and behaviors necessary to prevent diseases and improve health. | | | | | |
| 9 | Apply proper verbal and non-verbal communication skills to understand the supportive care demands of cancer survivors. | | | | | |
| 10 | Select and use the most effective method for communicating | | | | | |

| | | | | | | |
|---|---|---|---|---|---|---|
| | with cancer survivors. | | | | | |
| **( I can ...)** | | **1** | **2** | **3** | **4** | **5** |
| 11 | Verify if cancer survivors had accurately understood the education content and provide relevant feedback. | | | | | |
| 12 | Provide personalized education by considering the demand for education, literacy, language, and cultural influence of cancer survivors and their families. | | | | | |
| 13 | Cooperate with colleagues, doctors, and other healthcare providers to continuously provide integrated supportive nursing care for cancer survivors. | | | | | |
| 14 | Provide non-discriminating nursing care by respecting social and cultural diversities such as beliefs, customs, ethnicities, and religions of cancer survivors and families. | | | | | |
| 15 | Encourage cancer survivors and their families to express their demands and actively participate in management for recovery. | | | | | |
| 16 | Treat cancer survivors with a receptive attitude. | | | | | |
| 17 | Support autonomy and self-determination of cancer survivors. | | | | | |
| 18 | Identify and manage the factors hindering or promoting integrated supportive nursing care for cancer survivors. | | | | | |
| 19 | Improve the quality of nursing by evaluating the integrated supportive nursing care provided to cancer survivors. | | | | | |
| 20 | Define the performance goals of integrated supportive nursing care for cancer survivors. | | | | | |
| 21 | Contribute to the advancement of integrated supportive nursing care for cancer survivors by participating in relevant continuing education, license acquisition, publication, conferences, and presentations | | | | | |
| 22 | Apply nursing intervention based on newly discovered grounds by identifying the latest trends on integrated supportive nursing care for cancer survivors. | | | | | |
